# Percutaneous versus open cannulated screws fixation for displaced isolated medial malleolar fractures in adults: a randomized controlled clinical trial

**DOI:** 10.1007/s00402-025-06000-w

**Published:** 2025-07-25

**Authors:** Khalaf fathy elsayed Ahmed

**Affiliations:** Sohag University, Sohag, Egypt

**Keywords:** Isolated medial malleolar fractures, Percutaneous fixation, Open reduction, Cannulated screws, Randomized controlled trial

## Abstract

**Introduction:**

Isolated medial malleolar (MM) fractures are infrequent injuries. Studies focused on their treatment are scarce. The aim of this study was to compare functional and radiographic outcomes of two surgical techniques for treatment of displaced isolated MM fractures in adults: closed reduction and percutaneous fixation (CRPF), and open reduction and internal fixation (ORIF) by using same implant; two partially-threaded cannulated cancellous screws.

**Materials and methods:**

A prospective randomized controlled clinical trial (RCT) was conducted on 50 patients with isolated displaced MM fractures, treated with CRPF (group A) or ORIF (group B), at orthopaedics department of university hospital, from April 2021 to April 2023. Fractures were classified by Herscovici classification. The primary outcomes were incidence of complications and time to union based on radiographic assessment by plain radiographs of ankle. The secondary outcomes were functional assessment by Foot and Ankle Ability Measure (FAAM) for activities of daily living (ADLs) and sports, American Orthopaedic Foot and Ankle Society (AOFAS) score, and VAS.

**Results:**

No significant differences were noticed among two groups regarding age, sex, side affected, mechanism of injury, smoking, Herscovici classification, or follow-up duration. Mean final FAAM-ADLs was 97.6 ± 2 in group A, and 95 ± 3.4 in group B, *(P =* 0.155*)*. Mean final FAAM-sports was 87 ± 11.4 in group A, and 73.4 ± 15.6 in group B, *(P =* 0.312*)*. Mean final AOFAS score was 95.9 ± 8.4 in group A, and 94.6 ± 9.5 in group B, *(P =* 0.237*)*. Mean final VAS for pain was 0.9 ± 0.5in group A, and 1.5 ± 0.9 in group B, *(P =* 0.453*)*. Mean time of solid radiographic union was 9.5 ± 2 weeks in group A, and 10.4 ± 3 weeks in group B, *(P =* 0.026*)*.

**Conclusion:**

CRPF of displaced isolated MM fractures is an efficient method with comparable radiographic and functional outcomes to ORIF. Based on these results, percutaneous fixation could be a good alternative for managing displaced isolated MM fractures.

**Level of evidence:**

Level II therapeutic: Prospective randomized controlled clinical trial.

**Trial registration:**

The trial was registered at www.clinicaltrials.gov (Trial Registration Number: NCT06883435).

**Supplementary Information:**

The online version contains supplementary material available at 10.1007/s00402-025-06000-w.

## Introduction

Medial malleolar (MM) fractures in adults are intra-articular injuries that may occur in isolation or as a part of bi-malleolar, tri-malleolar, or quadri-malleolar associated ankle fractures. MM fractures as a part of associated ankle fractures are common injuries; however, isolated MM fractures are infrequent. The causes of their injuries are either motor car accidents, twisting of ankle, or falls [1–3].

Adequate management of MM fractures is essential for maintenance of ankle stability, normal biomechanics, and prevention of arthritis, varus malunion or non-union [4].

As a part of associated ankle fractures, MM fractures are typically treated surgically, however, there is no clear full consensus regarding the optimal treatment of isolated MM fractures due to their relative infrequency. Non-displaced isolated MM fractures can be treated non-operatively by below knee cast immobilization; however, displaced isolated MM fractures are usually treated surgically [5–8].

Different implants for fixation of MM fractures have been proposed depending on the size, shape, and comminution of fractured fragment including; malleolar screws, fully or partially threaded unicortical or bicortical compression screws, single or double screws, buttress plates, tension band wiring (TBW), headless screws, bio-absorbable magnesium screws or mini-screws [9–17].

MM fractures can be approached through traditional open reduction and internal fixation (ORIF), minimally invasive surgery (MIS), arthroscopic-assisted, or percutaneously [18–21]. ORIF allows adequate reduction and good fixation, however, it resulted in long scar, and high complication rates [22, 23]. Percutaneous fixation by cannulated lag screws is an effective method in recent years for many fractures.

There are many studies that had compared clinical and radiographic outcomes of using different methods of fixation and different surgical approaches for treatment of MM fractures (either in isolation or associated with bi- or tri-malleolar ankle fractures) [12, 17, 24], however, most studies focused on isolated MM fractures alone are scarce and focused only on comparison of osteosynthesis rather than the approach (open vs. percutaneous) [15, 25].

The objective of this prospective randomized controlled clinical trial (RCT) study was to compare the functional and radiographic outcomes between the percutaneous fixation and ORIF for the surgical treatment of displaced isolated MM fractures in adults using the same implant; two partially threaded 4 mm cannulated cancellous screws, so that the results can be attributed to the surgical approach used. The hypothesis was that CRPF would show comparable outcomes to ORIF.

## Materials and methods

### Trial design

This non-inferiority, parallel-group, prospective randomized controlled clinical trial study was conducted at the orthopedic and traumatology department of university hospital (tertiary level), from April 2021 to April 2023, after approval from the medical ethical committee of the faculty of medicine (IRB registration no: Soh-Med-23-03-09PD), and it was registered at ClinicalTrials.gov (NCT06883435). The study was carried out according to the Declarations of Helsinki, and informed written consents with risks explanation were obtained from all patients. The CONSORT guidelines for reporting RCTs were followed [26] (Supplementary material 1).

### Sample size

Sample size calculation by Open Epi program, version 3 open source calculator -SSCC using randomized clinical trials study mean difference equation when we depend on the previous results of Liu et al. [21]. The control numbers to case numbers were equal, within an error probability of 0.05 and 80% power on 2- tailed test (type 1 error). A type 1 error, a false positive result, occurs if the researcher rejects a true null hypothesis. A type 2 error, a false negative result, occurs if the researcher fails to reject a false null hypothesis. Calculating a reasonable sample size was needed to avoid the two types of errors. It was calculated that 16 patients in each group were needed. At the end of the study, 25 patients were collected in each group for final analysis.

### Participants (inclusion and exclusion criteria)

The inclusion criteria were adult patients (> 18 years) with closed, displaced (˃2 mm), isolated medial malleolar fractures of Herscovici type B or C, that were presented immediately after the injury (within 1–2 days). The exclusion criteria were; comminuted fractures, open fractures, bi-, tri-, or quadri-malleolar ankle fractures, associated syndesmotic or lateral collateral ligament injuries, skeletally immature patients, any other ipsilateral lower limb fractures, delayed presented fractures, or previously fractured ankle.

### Randomization and blinding

The patients were randomly allocated (allocation ratio 1:1) to one of the two treatment groups: group A; closed reduction and percutaneous fixation (CRPF), and group B; open reduction and internal fixation (ORIF) using a computer-generated system, and the details of random distribution were concealed in a sealed opaque envelopes. Patients were sequentially numbered and given to the head nurse, who informed the surgeon regarding the allocation before the operation. All surgeries were performed by the surgeon who was qualified to do both approaches. By default, surgeon couldn’t be blinded regarding the type of surgical intervention. However, the outcomes assessor was blinded to functional outcomes assessment by asking the patient to wear socks so that the assessor wouldn’t notice the surgical site.

### Interventions

#### Preoperative assessment

Pre-operative clinical assessment of the patients included; detailed history taking (age, gender, co-morbid conditions, medications and smoking status), general examination for detection of any associated injuries or fractures, and local examination of the fractured ankle for side of injury, swelling, and neurovascular condition. Routine laboratory investigations were done.

**Fig. 1:**
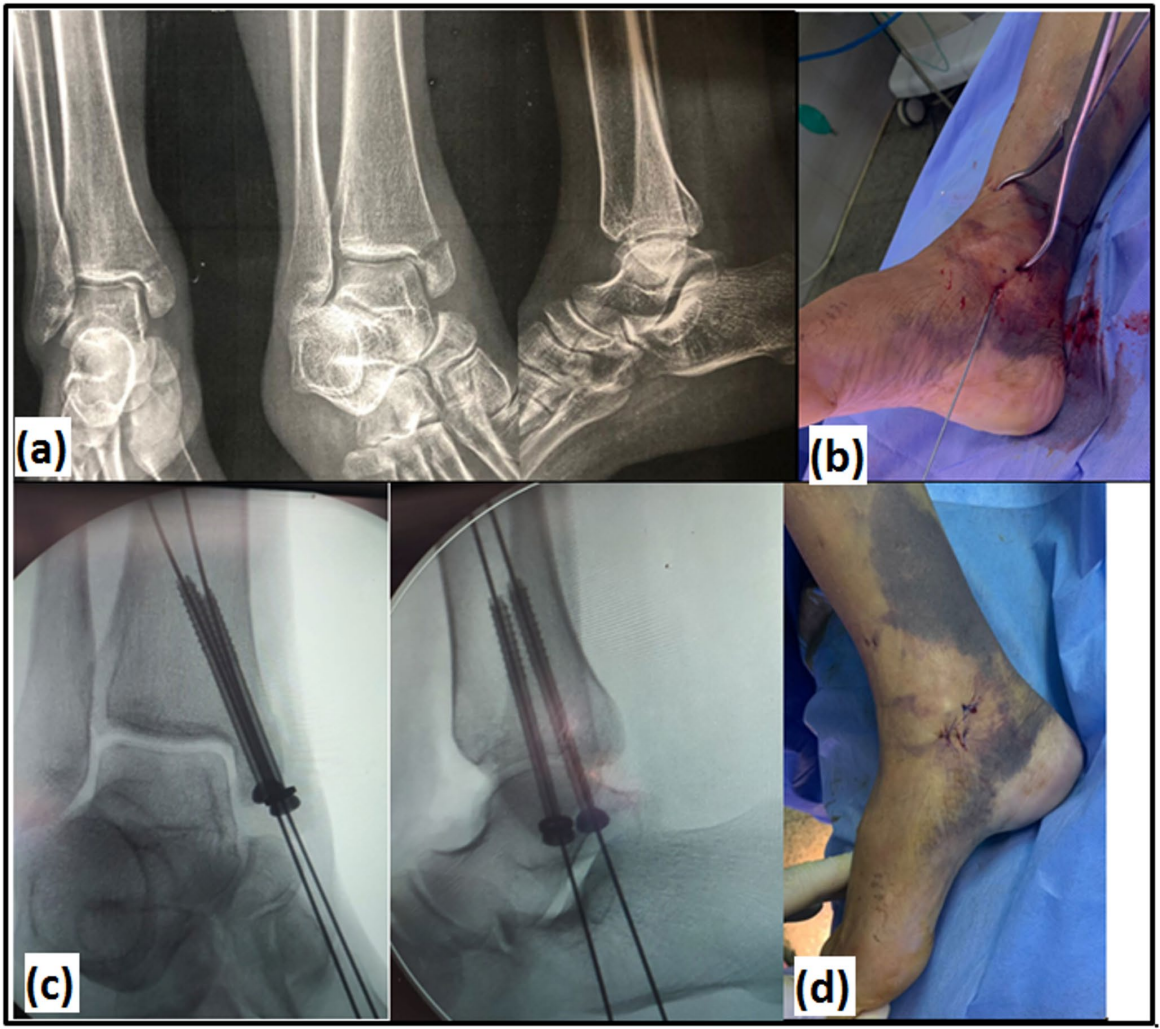
Surgical technique of CRPF: **a** pre-operative AP and lateral X-ray views of a patient with isolated MM fracture. Intra- operative photographs demonstrating; **b** holding the reduction by pointed reduction forceps applied from tip of MM perpendicular to fracture line, and then guide wires for cannulated screws were inserted one after another, **c** C-arm fluoroscopy showing cannulated screws insertion over guide wires **d** simple skin stitches for stab incisions

Radiographic evaluation consists of antero-posterior (AP), mortise, and lateral views of fractured ankle to allow preoperative classification of fractures. Plain radiographs of the whole leg were also obtained in some cases to exclude proximal fibular fractures and to rule out Maisonneuve injury (Figs. 1a, and 3a). Fractures were classified according to Herscovici classification [5].

**Fig. 2:**
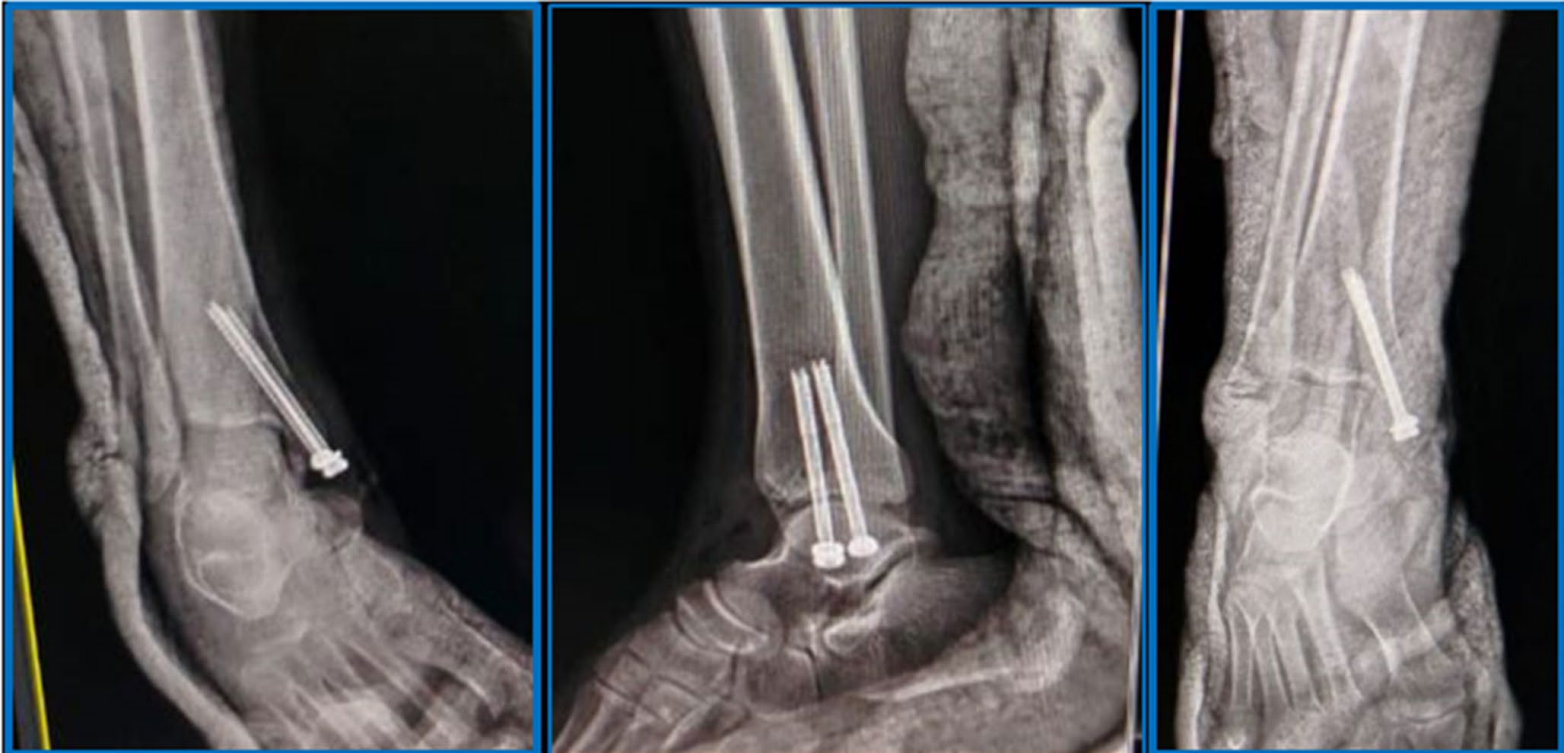
Surgical technique of CRPF (continued): post-operative radiographs

**Fig. 3:**
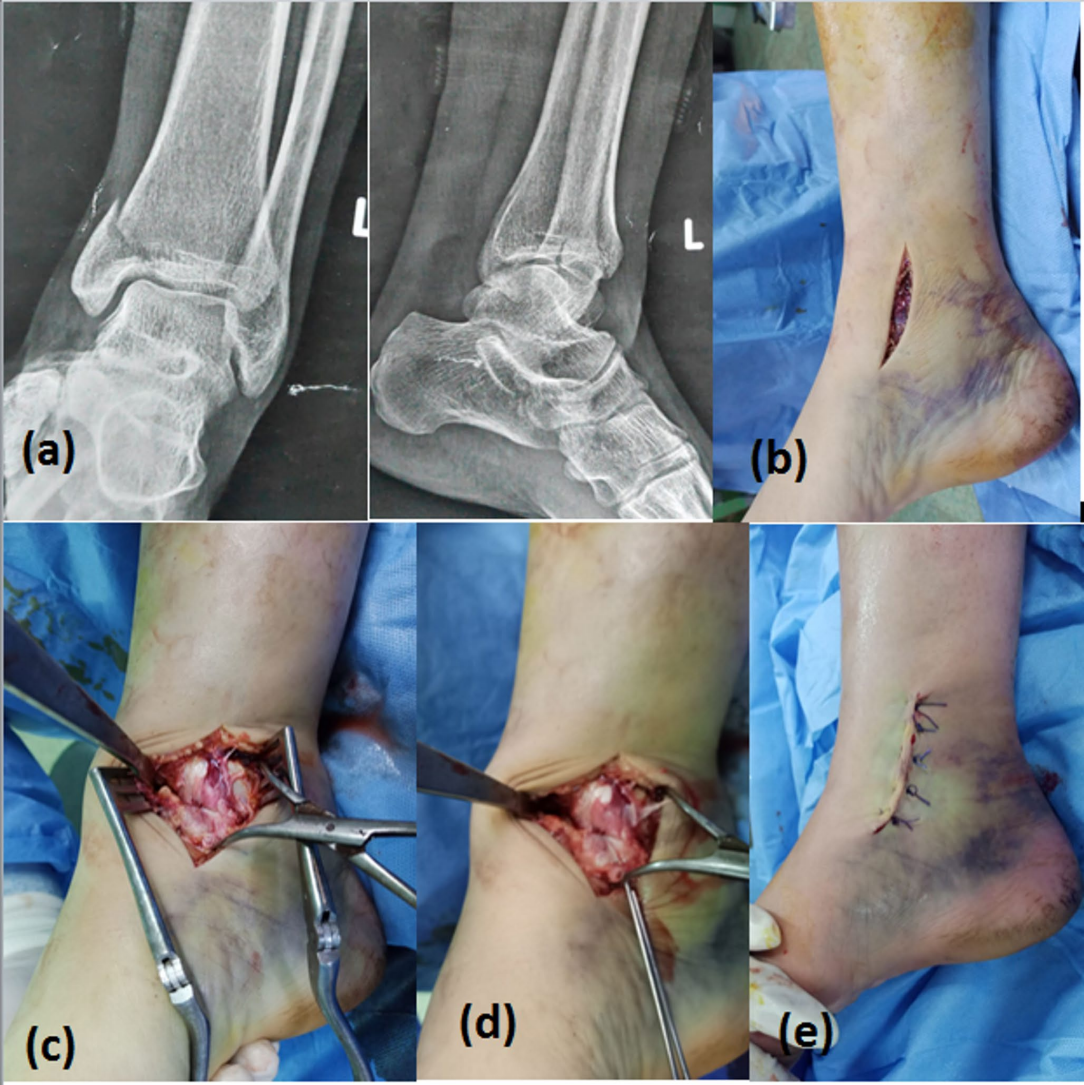
Surgical technique for ORIF: **a** pre-operative AP and lateral X-ray views of a patient with isolated MM fracture. Intraoperative photograph showing; **b** the open surgical approach, **c** pointed forceps holing the reduction, **d** inserting guide wires for cannulated screws, **e** skin closure by stitches

### Surgical techniques

Fractures were treated immediately after the injury (within 1–2 days). Operations were done under spinal anesthesia with patients were supine on radiolucent table, and guided by C-arm fluoroscopy for assessment of intra-operative reduction in both groups. The tourniquet was used in patients of ORIF group only.

####  Surgical technique of CRPF

No incision at the fracture site. Closed reduction of the fracture was done by traction, manipulation of the fractured ankle, and with help of pointed reduction forceps applied from the tip of medial malleolus perpendicular to the fracture line (Fig. 1b).

Through stab incisions, two partially threaded 4 mm cannulated cancellous screws with the use of washers were inserted over appropriate guide wires after drilling with appropriate drill bit for fixation of fracture under the guidance of C-arm fluoroscopy (Fig. 1c.)

The stability of ankle syndesmosis and lateral collateral ligament were checked after MM fixation by stress-testing of the ankle under anesthesia with fluoroscopic control. Finally, closure by simple stitches and below knee splint was applied for 2 weeks (Fig. 1d).

#### Surgical technique for ORIF

Curved antero-medial 3–5 cm incision was made over the medial malleolus (Fig. 3b). Debridement and reduction of the fracture under direct vision was done, reduction was held by pointed forceps applied from the tip of medial malleolus perpendicular to the fracture line (Fig. 3c). Fixation was done by two partially threaded 4 mm cannulated cancellous screws (Fig. 3d). Stability of syndesmosis and lateral collateral ligament were tested as in CRPF. Closure by simple stitches and below knee splint was applied for 2 weeks. (Fig. 3e)

### Post-operative care

**Fig. 4:**
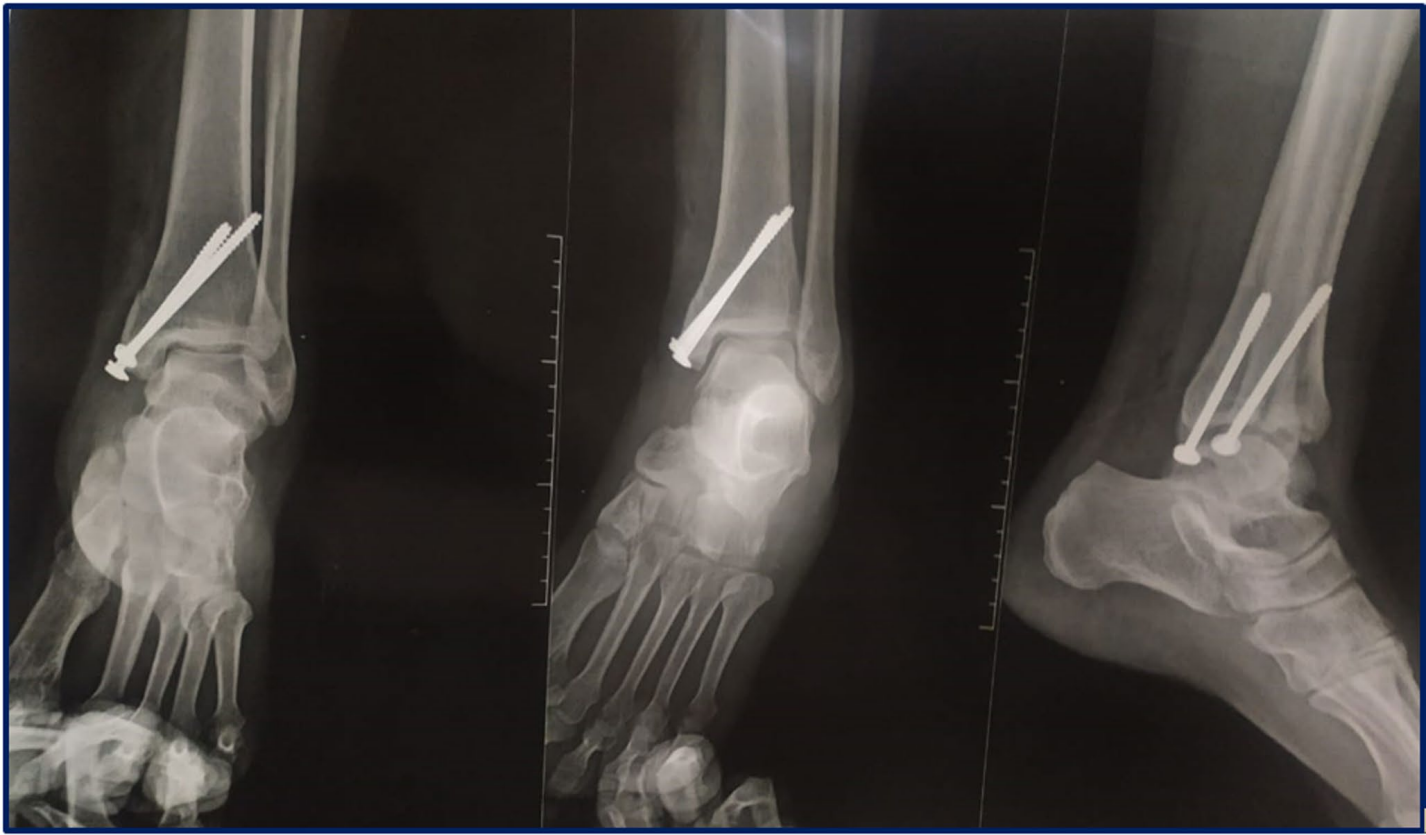
Surgical technique for ORIF (continued): post-operative radiographs

I.V antibiotics were given for the patients for three days post-operatively. Immediate postoperative AP, lateral, and mortise radiographs of the ankle were done to evaluate fracture reduction and fixation (Figs. 2 and 4).

### Outcomes assessment

Follow up at outpatient clinic was scheduled at: 2, 6, and 8 weeks, 3, 6, and 12 months. At 2 weeks post-operatively; stitches were removed and below knee cast was applied for another 4 weeks. At 6 weeks, cast was removed and weight bearing was started as tolerated.

**Fig. 5:**
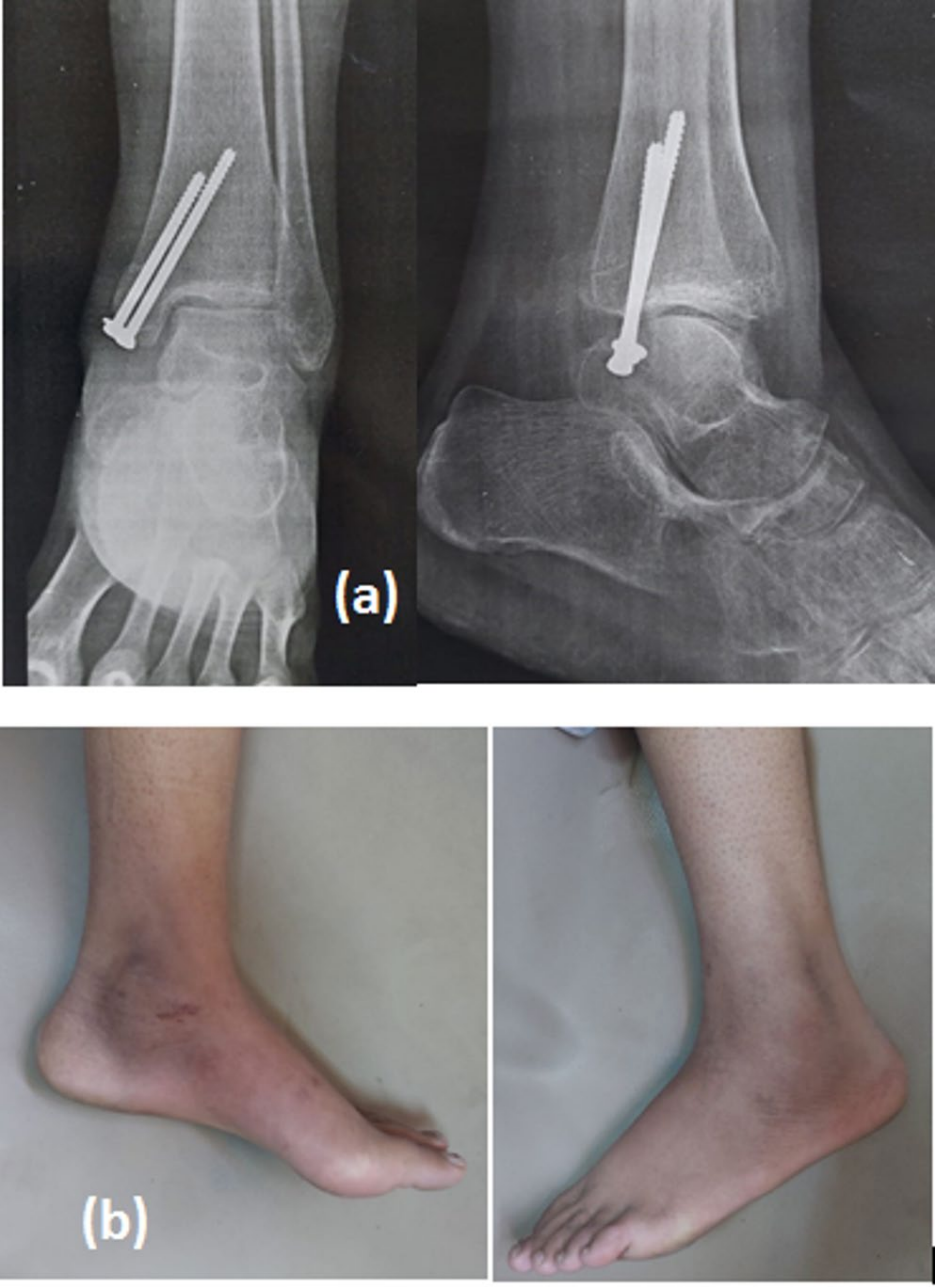
Follow-up: **a** radiographs showing complete union at final follow up. **b** Skin condition at final follow up showing minimal scar medially with no scars laterally

Clinical evaluations of patients at follow ups were done for pain, ankle range of motion (ROM), surgical site infection (presence of redness, serous or pus discharge), and any other appearing complications. Ankle radiographs (AP, mortise, and lateral) were done at follow up to assess fracture reduction, bone healing, and signs of OA in ankle joint. (Fig. 5a, b)

Fracture union was defined clinically as no tenderness at the fracture site on palpation or with ROM, and radiologically as obliteration of the fracture lines. Fracture nonunion was defined as failure to achieve these criteria at 6 months. Delayed fracture union was defined as absence of radiological progression of healing or instability of a fracture on clinical examination at 4–6 months after surgery.

Functional evaluation at final follow up was administered by outcomes assessor who was masked to the surgical site by asking the patients to wear socks. Functional outcomes were measured by Foot and Ankle Ability Measure (FAAM) for activities of daily living (ADLs) and sports subscales, American Orthopaedic Foot and Ankle Society (AOFAS) ankle-hindfoot score and VAS for pain [27–29].

### Statistical analysis

The collected data was computerized and statistically analyzed using SPSS program (Statistical Package for Social Science) version 25. Data was tested for normal distribution using the Shapiro-Wilk test. Qualitative data was represented as frequencies and percentages. Quantitative data was expressed as means and standard deviations. Student t-test and Mann–Whitney test were used for comparison of quantitative data between the two groups for parametric and non-parametric variables, respectively. Pearson Chi-square test or Fisher’s exact test were used for comparison of qualitative data between the two groups when appropriate. Level of P-value < 0.05 indicates significant while, *P* ≥ 0.05 indicates non-significant differences.

## Results

**Fig. 6:**
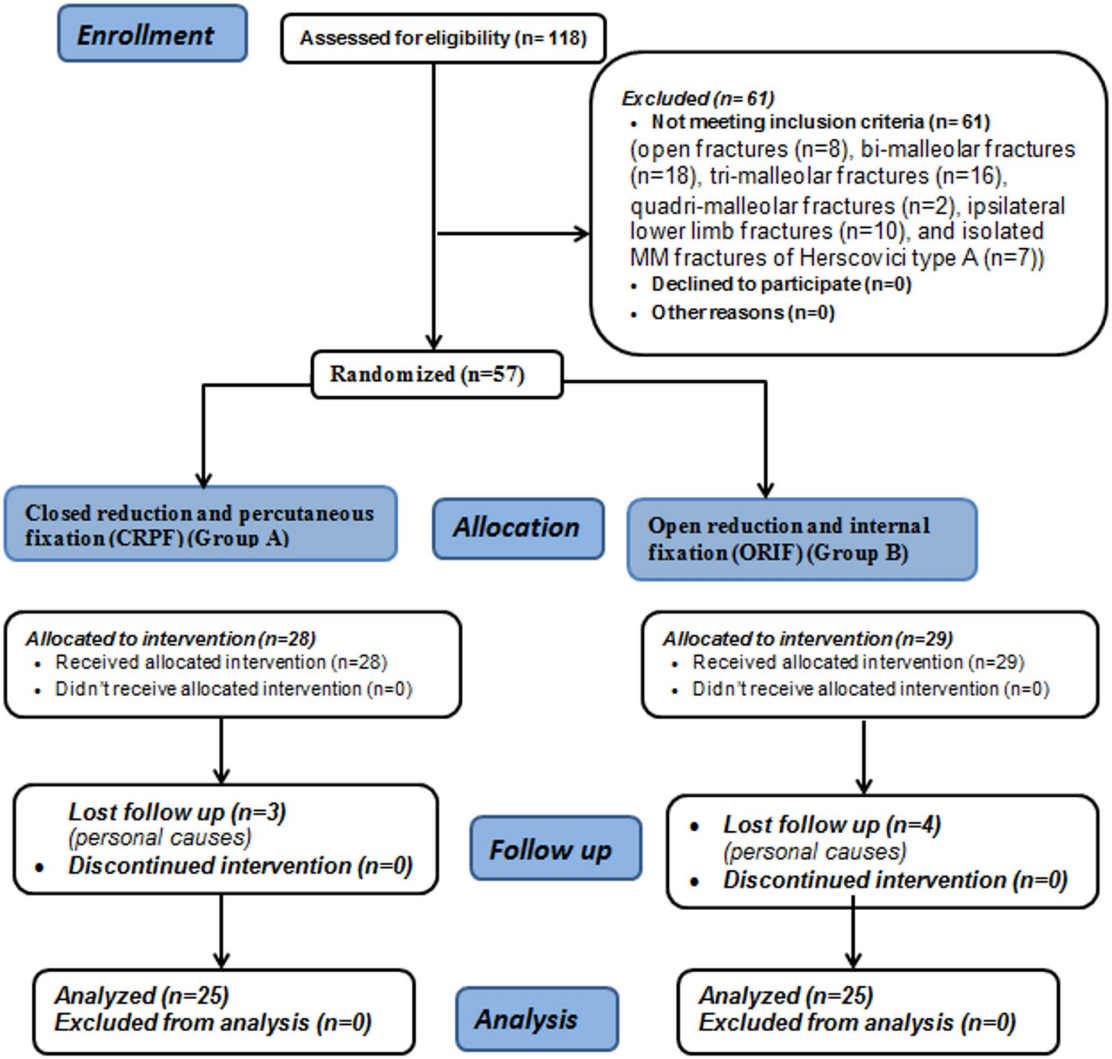
Consolidated Standards of Reporting Trials (CONSORT) flow diagram of the patient’s enrollment

118 patients were assessed for eligibility throughout the study period, of them 61 patients didn’t meet the criteria for inclusion (open fractures (*n* = 8), bi-malleolar fractures (*n* = 18), tri-malleolar fractures (*n* = 16), quadri-malleolar fractures (*n* = 2), ipsilateral lower limb fractures (*n* = 10), and isolated MM fractures of Herscovici type A (*n* = 7)), and only 57 patients were eligible for inclusion (28 patients were treated by CRPF and 29 by ORIF). Seven patients were lost during follow up due to personal causes (in group A (*n* = 3), and in group B (*n* = 4)), so 50 patients with displaced (˃2 mm) isolated MM fractures were left for final analysis (25 in each group), the details of allocation are shown in Fig. 6.

No significant differences were noticed among the two groups regarding age, sex, side affected, mechanism of injury, smoking, Herscovici classification, time between injury and surgery, or follow up duration. The operative time was 40 ± 5 (35–50) min and 60.5 ± 6.7 (50–75) min for group A and group B, respectively, (*P* =  0.042) (Table 1).

**Table 1:** Demographics and basic variables of the two groups

Variable	Group A: CRPF	Group B: ORIF	P-value
Total patients	25	25	
Age (years)			
Mean ± SD (Range)	36 ± 9.6 ( 18–63)	37 ± 12 ( 19–67)	0.175
Sex, no (%)			
Males	13 (52%)	15 (60%)	0.963
Females	12 (48%)	10 (40%)	
Side affected, no (%)			
Right	11 (44%)	12 (48%)	0.672
Left	14 (56%)	13 (52%)	
Mechanism of injury, no (%)			
Sports participation injury	7 (28%)	9 (36%)	0.482
Falling down stairs	10 (40%)	7 (28%)	
Motor car accident	8 (32%)	9 (36%)	
Herscovici classification, no (%)			
Type B	15 (60%)	11 (44%)	0.081
Type C	10 (40%)	14 (56%)	
Smoking, no (%)			
Non-smokers	14 (56%)	13 (52%)	0.742
Smokers	11 (44%)	12 (48%)	
Time delay until surgery (days)			
Mean ± SD (Range)	1 ± 0.5 (1–2)	1 ± 1.5 (1–3)	0.07
Operative time (minutes)			
Mean ± SD (Range)	40 ± 5 (35–50)	60.5 ± 6.7 (50–75)	**0.042 (S)**
Follow up period (months)			
Mean ± SD (Range)	16.56 ± 3.7 (12–24)	17.3 ± 3.86 (12–24)	0.844

### Functional outcomes

At the final follow up; the mean final FAAM-ADLs was 97.6 ± 2 (65–100) in group A, and 95 ± 3.4 (45–100) in group B, (*P* = 0.155). The mean final FAAM-sports was 87 ± 11.4 (35–100) in group A, and 73.4 ± 15.6 (30–100) in group B, (*P* = 0.312). The mean total AOFAS ankle-hindfoot score was 95.9 ± 8.4 (70–100) in group A, and 94.6 ± 9.5 (70–100) in group B, (*P* =  0.237). There were 12 (48%) patients had excellent scores, 11(44%) patients had good scores, and two (8%) with fair scores in group A, and there were 11 (44%) patients had excellent scores, 11 (44%) patients had good scores, and three (12%) with fair scores in group B, with no poor scores in both groups, (*P* = 0.316). The mean final VAS score for pain was 0.9 ± 0.5 (0–2) in group A, and 1.5 ± 0.9 (1–3) in group B, (*P* = 0.453) (Table 2).

**Table 2:** Radiographic and functional outcomes of the two groups

Outcomes		Group A: CRPF	Group B: ORIF	P-value
Time of radiographic solid union (weeks)				
Mean ± SD (Range)		9.5 ± 2 (8–17)	10.4 ± 3 (8–19)	**0.026 (S)**
Number of screws used				
		2	2	1.0
Foot and ankle ability measure (FAAM) at final follow up, Mean ± SD (Range), (%)				
FAMM-ADLs		97.6 ± 2 (65–100)	95 ± 3.4 (45–100)	0.155
FAAM-sports		87 ± 11.4 (35–100)	73.4 ± 15.6 (30–100)	0.312
AOFAS ankle-hindfoot score at final follow up (points)				
Mean ± SD (Range)		95.9 ± 8.4 (70–100)	94.6 ± 9.5 (70–100)	0.237
Excellent, no (%)		12 (48%)	11 (44%)	0.316
Good, no (%)		11(44%)	11 (44%)	
Fair, no (%)		2 (8%)	3 (12%)	
Poor, no (%)		0 (0.0%)	0 (0.0%)	
VAS score for pain (points) at final follow up=				
Mean ± SD (Range)		0.9 ± 0.5 (0–2)	1.5 ± 0.9 (1–3)	0.453
Complications				
Superficial infections		0 (0%)	3 (12%)	**0.032 (S)**
Deep infections		0 (0%)	0 (0%)	
Symptomatic implants		1 (4%)	1 (4%)	
Ankle arthritis		0 (0%)	0 (0%)	

### Radiographic outcomes

Two partially threaded cannulated cancellous screws were used for each patient. All fractures were healed before 6 months without any displacement, malunion, nonunion or implant failure. The mean time of solid radiographic union was 9.5 ± 2 (8–17) weeks in group A, and 10.4 ± 3 (8–19) weeks in group B, (*P* = 0.026) (Table 2).

### Complications

Post-operative complications occurred for four patients in group B; Three (12%) patients developed superficial infections which were treated by daily dressings and antibiotics, and one (4%) patient had symptomatic implant, however in group A only one (4%) patient had symptomatic implant, (*P* = 0.032) (Table 2).

## Discussion

After a follow up period of 16.56 ± 3.7 (12–24) months in percutaneous fixation (CRPF group), and 17.3 ± 3.86 (12–24) months in ORIF group, the major finding of this RCT study was no clinical or radiographic superiority of ORIF over percutaneous fixation for treatment of displaced isolated MM fractures in adults.

Different types of osteosynthesis have been proposed for internal fixation of displaced isolated MM fractures. In the current study; two partially threaded 4 mm cannulated cancellous lag screws were used for each case in both groups as screw fixation is one of the most commonly accepted standard construct for MM fractures stabilization and can be used in both techniques of percutaneous fixation and ORIF to avoid confounding factors that affect the results if different implants were used, and thus the outcomes could be attributed to surgical approaches [9, 24]. Two partially threaded cannulated cancellous screw added more compression than one screw only. In addition, cannulated partially threaded 4 mm cancellous screws are more cheaper than some fully threaded screws especially headless ones, and previous studies demonstrated no superiority of fully threaded over partially threaded screws with both had good and comparable clinical results [30, 31].

Different tools can be used for measuring clinical outcomes of various ankle conditions including; Patient-Reported Outcomes Measurement Information System (PROMIS) [32], FAAM [27, 33], Foot and Ankle Outcome Score (FAOS) [34], Short Musculoskeletal Function Assessment (SMFA) [35], and AOFAS score [28]. The clinical outcomes in this study were evaluated by FAAM-ADLs and sports subscales as it is proven to be reliable instrument for assessing foot and ankle disability, and effective for clinical assessment and research within Arabic-speaking populations [33]. However, AOFAS score was discouraged by AOFAS as a sole instrument for reporting outcomes of different foot and ankle problems [36], it was used in this study in addition to FAAM to allow for comparison of the results of the study with previous studies.

Regarding functional outcomes; there were no statistically significant differences between the two groups regarding FAAM, AOFAS score, or VAS at the final follow up. The functional outcomes of the current study were comparable to other studies for isolated MM fractures alone or associate with bi- or tri-malleolar fractures.

There are many studies that had compared clinical and radiographic outcomes of treatment of MM fractures (either in isolation or associated with bi- or tri-malleolar ankle fractures). Matson et al. [24], retrospectively reviewed records of patients with MM fractures either isolated or bimalleolar types treated with CRPF or traditional ORIF, and found that no statistically significant difference between the CRPF and ORIF groups regarding complication rates or time to full union at 8.9 ± 4 weeks and 9.6 ± 12 weeks, respectively (*P* = 0.78). May et al. [17], retrospectively compared clinical and radiographic outcomes of MM fractures (either isolated or associated with bi- or tri-malleolar ankle fractures) fixed with either bio-absorbable magnesium screws or conventional titanium screws, the clinical results were assessed by AOFAS score, and found that both methods of fixation had similar safety and therapeutic efficacy, however, use of magnesium screws prevent need for implant removal but more expensive in cost, and thus they recommended bio-absorbable magnesium screws for fixation of MM fractures. Mohammed et al. [12], prospectively compared two methods of internal fixations of MM fractures (either isolated or associated with bi- or tri-malleolar ankle fractures) treated by either malleolar screw or TBW, and found that TBW was better than screw fixation.

Previous studies focused on treatment of isolated MM fractures alone are scarce, retrospective, and focused only on comparison of different implants for fixation without giving concern to comparison of surgical approaches. Aydin and Çınaroğlu [25], retrospectively reviewed results of 23 patients with isolated MM fractures treated by 4-mm lag screw and Kirschner wire (K-wire) versus 4-mm lag screw, K-wire, and anti-gliding mini-plate for extra-stability, the results were assessed by AOFAS score and VAS for pain, and concluded that additional mini-plate had better early outcomes (at 2nd month post-operatively) with earlier return to daily life activities, however, both techniques have similar mid-term outcomes (at 6th, 12th, and 24th months post-operatively). Bulut and Gursoy [15], retrospectively compared the clinical and radiographic results of 32 patients with isolated MM fractures who were surgically treated by different fixation methods either headless cannulated fully threaded compression screws or conventional techniques (cancellous lag screws or TBW), after follow up periods of 12–55 months, the clinical results were assessed by AOFAS score and VAS for pain, and reported satisfactory results with the three fixation methods, however, pain on palpation of MM due to implant irritation was lower with using headless cannulated fully threaded compression screws, and thus they recommended headless cannulated fully threaded compression screws for fixation of isolated MM fractures.

Solid radiographic union of all fractures was obtained without malunion at statistically significant shorter time in CRPF (group A) at 9.5 ± 2 (8–17) weeks compared to ORIF (group B) at 10.4 ± 3 (8–19) weeks, (*P* = 0.026). Compression produced at the fracture site by two partially threaded cancellous screws can overcome the deleterious effect of periosteum which could be introduced into the fracture site. Percutaneous fixation avoids long incision, extensive periosteal stripping, and damage of blood supply to the fracture site that occur with ORIF leading to prolonged fracture healing. The results of the current study don’t support the theory that CRPF could result in unacceptable reduction and delayed healing due to periosteal interposition at fracture site.

The operative time was significantly shorter in CRPF group A at 40 ± 5 (35–50) min compared to ORIF group B at 60.5 ± 6.7 (50–75) min, (*P* =  0.042*)*. Yañez Arauz [20], reported mean duration of the ankle surgery with MIS approach was 32.8 min.

Regarding the complications in the current study; there were no severe complications as deep infections, ankle arthritis, non-union, or malunion; however, minor complications were observed [37]. In ORIF (group B), superficial infections were observed in three cases (12%), and were treated by sterile dressings and antibiotics. Symptomatic implants were observed in one patient (4%) in each group, and were managed by removal, *(P =* 0.032). Pilskog et al. [23] reported the prevalence of fracture-related infection in patients with surgically treated ankle fractures as 9%.

The main strength of this prospective RCT study is the comparison of the surgical approaches (open vs. percutaneous) for similar isolated MM fractures in adults treated by similar osteosynthesis; two partially threaded 4 mm cannulated cancellous screws. It reported percutaneous fixation could be a good alternative to ORIF for managing displaced isolated MM fractures in adults.

The limitations of this study are; being single center study, relatively small sample size, relatively short follows up duration, lack of use of weight-bearing CT scan, and lack of measurement of the difference in radiation exposure between both approaches. In addition, percutaneous fixation without debridement of fracture hematoma is only feasible within the first 1–2 days of the injury, and that this technique may not generalizable if fractures are fixed 7–14 days after injury.

Multicenter studies comparing ORIF, percutaneous fixation, and arthroscopic-assisted fixation of isolated MM fractures in adults with long term follow ups, and based on ankle weight-bearing CT scan for analysis should be conducted in the future.

## Conclusion

No clinical or radiographic superiority of ORIF over percutaneous fixation for treatment of displaced isolated MM fractures in adults. Percutaneous fixation had the advantages of shorter operative time and lower complication rates. Based on these results, percutaneous fixation could be a good alternative for managing displaced isolated MM fractures.

## Electronic supplementary material

Below is the link to the electronic supplementary material.

Supplementary Material 1

## Data Availability

The datasets generated and/or analyzed during the current study are available from the corresponding author on reasonable request.
