# Correction: Percutaneous versus open cannulated screws fixation for displaced isolated medial malleolar fractures in adults: a randomized controlled clinical trial

**DOI:** 10.1007/s00402-025-06055-9

**Published:** 2025-09-08

**Authors:** Khalaf fathy elsayed Ahmed

**Affiliations:** Sohag University, Sohag, Egypt

**Correction: Archives of Orthopaedic and Trauma Surgery (2025) 145:385**

10.1007/s00402-025-06000-w

In the original version of this article, Figs. 5 and 6; Table 1 captions were mistakenly repeated under declaration section and these captions should have been removed.

The original article has been corrected.

